# Safety and efficacy of the new modified technique for c2 nerve root resection in 3d fluoroscopy navigated instrumentation in the cranio-cervical junction

**DOI:** 10.1007/s00701-024-06265-x

**Published:** 2024-09-16

**Authors:** Lukas Bobinski, Linda Sandberg, Frida Bylander, Hampus Hållberg, Anders Berglund, John M. Duff

**Affiliations:** 1 Department of Orthopedics, Spine Section, University Hospital Umeå, Umeå, Sweden; 2 Department of Diagnostics and Intervention, Orthopedics, Umeå University, Umeå, Sweden; 3 Department of Cardiology, Sunderbyn Hospital, Luleå, Sweden; 4 Epistat AB, Statistical Analysis, Uppsala Science Park, Uppsala, Sweden; 5 I3Spine International, Vaud, Switzerland

**Keywords:** Cranio-cervical junction, C2 nerve root ganglion, Radiculopathy, Neurectomy, Occipital neuralgia

## Abstract

**Purpose:**

Instrumentation of the C1 vertebra requires either mobilization or transection of the C2 nerve root. This study investigates clinical and radiological outcomes and incidences of C2 neuropathic pain after posterior instrumented fusion in the cranio-cervical junction with or without division of the C2 nerve roots.

**Methods:**

This retrospective study compared two cohorts of patients who underwent instrumented fusion in the cranio-cervical junction. Fifty patients (22 males and 28 females) were operated with complete resection of C2 nerve root ganglion (Ex group), and fifty-one patients (30 men, 21 women) with C2 nerve roots preservation (No group).

**Results:**

The incidence of postoperative C2 neuropathy was eight times lower in the Ex group compared to the No group that was statistical significant, *p* = 0.039. Surgical time was significantly shorter in the No group (*p* = 0.001). The fusion rates were very high for both groups, without difference between groups (*p* = 1.0). Autografting from the iliac crest (*p* = 0.001) as well as postoperative immobilisation with a hard collar (*p* < 0.001) were required in fewer patients in the Ex group. Also, patients in the Ex group were mobilised faster after surgery (*p* = 0.49). Overall, complication rates were similar between groups, but the Ex group demonstrated fewer major medical complications (16% vs 31%). Male sex and iliac bone harvesting demonstrated significantly higher OR for development of postoperative complications (*p* = 0.023 and *p* = 0.034 respectively) and postoperative mobilization demonstrated significant higher OR for development of postoperative major complications (*p* = 0.042).

**Conclusions:**

Resection of the C2 nerve root ganglion during posterior instrumented fusion of the cranio-cervical junction is safe and rarely leads to C2 neuropathy. The technique tends to mitigate the odds of developing postoperative complications.

**Supplementary Information:**

The online version contains supplementary material available at 10.1007/s00701-024-06265-x.

## Introduction

The cranio-cervical junction is a complex region of the cervical spine with close anatomical relationship between the skull base, C1 and C2 vertebrae, spinal cord, upper cervical nerve roots, and the vertebral arteries. Surgical treatment of different pathologies in this area can be technically demanding. Possible injury to vertebral arteries or the spinal cord during dissection, decompression and/or instrumentation can have a devastating impact on the outcome of patients. The most common surgical stabilization technique used in cranio-cervical junction is posterior instrumentation with screws and rods. In 1994, Goel and Laheri described a technique in which transection of the C2 nerve root provides improved access to the lateral mass of the C1 vertebrae for screw placement, which is then connected to the C2 screw by a plate [11]. The technique described involves opening of the C1/C2 joint space, removal of articular cartilage, and with bone graft placement bilaterally in the C1/C2 joints. This eliminates the need for midline wiring techniques with a posterior graft between the posterior elements of the C1 and C2 vertebrae for bone fusion. Excision of the C2 nerve root has many advantages such as a clear visualisation of the anatomy of C1 lateral masses, exposure and access to C1/C2 joints for arthrodesis, and less perioperative bleeding [10, 18, 32]. Harms and Melcher described a similar technique that requires placement of four screws, two in C1 lateral masses and two in C2 vertebra connected by rods without transection of the C2 nerve root [14]. Hence, to access the lateral mass of C1, the C2 nerve root needs to be mobilized and displaced caudally during C1 screw placement.

At our Spine Unit (Department of Orthopedics at Umeå University Hospital), image guided posterior instrumented fusions combined with intentional bilateral C2 nerve root ganglion resection, and with bone graft placement within the C1-C2 facet joint space has been used consecutively to treat most severe pathologies in the cranio-cervical junction. Our clinical experience suggests that this technique contributes to good postoperative pain control and induces solid fusion without the necessity for tricortical iliac crest grafting between the posterior elements of C1 and C2. Postoperative hypesthesia in the occipital region has not been observed to be a clinical problem, and if present patients tend to ignore it.

The goal of this study was to compare the clinical and radiological outcomes in two cohorts – the first one operated with C2 nerve-root ganglion excision (Ex) and the second with C2 nerve-root preservation (No), and to investigate whether there are differences in postoperative recovery time, complication rates and pain control.

## Materials and methods

After receiving ethical board approval, data were obtained from all patients operated 2014—2022 with a posterior instrumented fusion incorporating C1vertebrae fixation. Digital medical and operative records (System Cross, Orbit, Pin-Point) were used to extract demographic data as well as perioperative- and postoperative data for evaluation of the surgical and clinical outcomes that included neurological status, cervical pain, presence of C2 neuropathy and hypesthesia in the occipital region. All radiological images were evaluated using an image viewing software (SECTRA). Analysis of clinical outcome included perioperative and postoperative complications, mobilization time, use of a hard collar postoperatively, neurological status, and the presence of C2 neuropathy. Complications were defined as an adverse event, medical or surgical, appearing perioperative or within six weeks after the surgery, which was not due to underlying disease. These were described as major, minor or none. A major complication was defined as an adverse event causing either permanent detrimental effect or death or requiring re-operation. A minor complication was defined as an adverse event causing only transient detrimental effect [20, 28]. Wound infection was analysed separately, and was defined as occurring within six weeks postoperatively. Mobilization was described as a patient standing and/or walking or not mobilized at all. Neurological status was investigated using the American Spinal Injury Association score (ASIA score) pre- and postoperatively [25]. Neck pain was considered low if the numeric rating scale (NRS) was 1–4, medium if NRS was 5–7, and severe if NRS was 8–10. Pain described by patients as negligible was defined as a low NRS score. C2 neuropathy was defined as the presence of severe, unpleasant, neuropathic pain with sensory disturbance with or without dysesthesia within the C2 dermatome. Information regarding hypesthesia in the C2 innervated area was collected at the final follow-up. Incidence of postoperative C2 neuropathy, paraesthesia, and hypesthesia were also evaluated from medical records. All patients underwent surgery with an image-guided screw placement by using the O-arm™ in combination with the Stealth-Station™ (Medtronic).

Inclusions criteria were:

- Age ≥ 18 years old at the time of the surgery
- Instrumentation of C1 vertebra with a screw implanted in the lateral mass according to either Goel-Laheri or Harms-Melcher techniques.
- Patients with C2 nerve root ganglion resection were included regardless of whether the C1 vertebrae was instrumented or not.

Exclusion criteria were:

- Patients younger than 18 years old at time of surgery.
- Surgery without instrumentation of C1 vertebrae.
- Instrumentation without navigation.
- Patients who were treated with C2 nerve-root preservation and instrumentation of C1 vertebrae that does not require manipulation of the C2 nerve root (translaminar screws, hooks, wiring).
- Patients operated with only transection of C2 nerve roots.

### Surgical technique for C2 nerve root ganglion excision and navigation

After careful intubation patients were placed in a prone position on a Jackson table with the cervical spine secured using a Mayfield head holder. A midline incision was conducted from the skull base to the level of the lowest instrumented vertebrae. Meticulous exposure of the posterior elements was followed by microsurgical exposure of the C2 nerve root ganglia. The venous plexus surrounding the C2 ganglion on each side was then coagulated, dissected, and divided sharply. Oozing from the plexus was stopped by using bipolar cautery, a haemostatic agent (Surgiflow®), and a microsurgical cotton patty under gentle suction. The ventral and dorsal roots of the C2 nerve root were exposed both proximal and distal to the ganglion. The ganglion was then resected. Close attention was paid to avoid any cerebro-spinal fluid (CSF) leak and/or injury to the vertebral artery (VA) that is usually located just lateral to the C2 nerve-root after it divides into ventral and dorsal branches. The C1/C2 joints were then easily visible and accessible. After opening the joint capsules, cartilage was removed, and joints were decorticated using a high speed burr (Midas Rex™) and micro-curettes. Screws were then placed using a navigation (O-arm™) with reference frame placed toward the head, and attached to the C2 spinous process. The position of the implants was verified using a post implantation intraoperative 3D image acquisition.

All of the surgeries in the Ex group were performed by the senior author (LB) with complete excision of the C2 nerve root ganglion.

### Surgical technique for C2 nerve root preservation and navigation

The decision about C2 nerve root preservation was at the discretion of the surgeon. Like the above described procedure, patients in the No group were carefully intubated and placed in a prone position on a Jackson table with the cervical spine secured using either a Mayfield head holder or Gardner-Wells traction device. A midline incision was conducted from the skull base to the level of the lowest instrumented vertebrae. Meticulous exposure of the posterior elements was followed by microsurgical exposure of the C1 arch, venous plexus surrounding C2 nerve roots, as well as the C2 vertebrae. The mobilisation of the C2 ganglion was executed in the subperiosteal at the junction between the C1 lateral mass and the C1 arch. The nerve was pulled caudally to expose the starting point for C1 instrumentation. Bleeding from the plexus was stopped by using a haemostatic agent (Surgiflow®) and a piece of gelatine absorbable sponge (Songostan™) along with a short application of microsurgical cotton patty under gentle suction. Similar to the Ex group, screws were then placed using navigation (O-arm™) with reference frame placed toward the head, and attached to the C2 spinous process. The position of the implants was verified using a post implantation intraoperative 3D image acquisition.

### Clinical and radiological follow-up criteria

All included patients were followed clinically and radiologically. Due to long geographical distances within our region and advanced age of the majority of the patients, most of the clinical follow-ups were conducted via a telephone call preferably scheduled at 3, 6 and 12 months after the surgery. If the patients reported any signs of complication, they were clinically evaluated, and if necessary, re-operated at our clinic. Computer tomography (CT) was performed to evaluate postoperative fusion, which was defined as any callus between the operated vertebrae or/and within the fracture.

### Statistical analysis

Descriptive variables are presented as continuous or categorical groups based on the nature of the distributions by surgical intervention. Continuous data are presented as median with inter quartile ranges (IQR). The Mann–Whitney U test or the chi-square test was used to assess differences between the surgical interventions. Univariable logistic regression models were used to estimate the odds ratio with the corresponding 95% confidence intervals (95 CI) with complications as the outcome and for pre-specified variables of interest. In a first step, outcomes were defined as minor and major complications, and then restricted to only major comparisons. A statistically significant two-sided P-value was < 0.05. Microsoft Excel was used to store raw data, and all data analysis and statistical analyses were performed using the statistical program R version 4.3.0.

## Results

One hundred and eighty-six medical records of the patients operated in the cranio-cervical junction were revised during enrolment in the study. One hundred and one patients met the inclusion criteria and were enrolled in two cohorts. Fifty patients (22 males, 28 females) that were operated with excision of the C2 nerve-root ganglion were enrolled to the excision group (Ex). Fifty-one patients (30 males, 21 females) with preserved C2 nerve roots were enrolled in the control group (No). Direct comparison confirmed that two groups were well-matched regarding age, sex, clinical follow-up, and preoperative American Society of Anaesthesiologist classification (ASA). However, there was a significant difference regarding two variables. The patients within the Ex group were surgically treated for more complex pathologies that included pseudarthrosis (8%), deformities like basilar impression/invagination (14%), rheumatoid arthritis (RA) (22%), and tumours (8%) (*p* < 0.001). Also, radiological follow-up in the Ex group was significantly shorter (*p* = 0.015). Detailed information about both groups regarding sex, age, ASA score and indication for surgery is described in Table 1. Moreover, the extent of cervical fixation was similar in both groups (Table 2).

**Table 1:** Demographical characteristics by surgical interventions

	Ex group	No group	P-value	Total
	No. Of Pts (%)	No. Of Pts (%)		No. Of Pts (%)
All subjects	50 (100.0)	51 (100.0)		101 (100.0)
Sex			0.2	
Female	28 (56)	21 (41.2)		49 (48.5)
Male	22 (44)	30 (58.8)		52 (51.5)
Age at surgery, median [IQR]	71.50 [61.25, 78.0]	73.0 [66.0, 78.0]	0.6	73 [64.0, 78.0]
ASA classification			0.1	
1	1 (2)	6 (11.8)		7 (6.9)
2	18 (36)	18 (35.3)		36 (35.6)
3	34 (60)	23 (45.1)		53 (52.5)
4	1 (2)	4 (7.8)		5 (5.0)
Indications			< 0.001	
Trauma	19 (38) **	44 (86.3) **		63 (62.4)
Other	31 (62) **	7 (13.7) **		38 (37.6)
Clinical follow-up, median [IQR]	12.0 [7.75, 16.25]	13.0 [7.5, 26.5]	0.4	12.0 [7.75, 22.0]
Radiological follow-up, median [IQR]	11.0 [7.0, 21.75] *	18.0 [11.0, 32.0] *	0.015	15.0 [7.00, 28.0]

*p < 0.05, Mann Whitney U test **, *p* < 0.01, chi-square test for heterogeneity

Pt = patient

**Table 2:** Type and extent of cervical fixations

Fixation type	EX (50)	NO (51)
C0-subaxial*	3	1
C0-C2/C3*	3	2
C1/C2	33	26
C1-C3	4	12
C1	1	3
C1-subaxial	6	7

^*^ The construct includes instrumentation of C1 lateral masses with screws

### Clinical outcome

Patients in the Ex group required significantly longer surgery (*p* = 0.001) but had less perioperative bleeding than patients in the No group (*p* = 0.072). Instrumentation to the occiput was performed in 11% of the Ex group, and in 6% of the No group. Minor complication rates were the same in both groups. On the other hand, major complications were more common in the No group compared to the Ex group (31% vs 16%); but overall complications rates were not significantly different between groups (*p* = 0.184). Also, there were no significant differences between groups regarding infection rate, and re-operation at 3 months and 1 year, respectively.

The neurologic status pre- and postoperatively in both groups, according to the ASIA score, was not significantly different (*p* = 0.612). Yet, 8% of the patients in the Ex group improved compared to the No group (2%), but this did not reach statistical significance (0.347). There were no neurological deteriorations in either group after surgery.

The Ex group required significantly less iliac bone harvesting (*p* = 0.001), were mobilised sooner (*p* = 0.049), and did not required utilization of cervical collar in the postoperative period (p < 0.001) compared to the No group. There was no difference in cervical pain between the groups at the last follow-up (*p* = 0.315). The Ex group had a lesser incidence of C2 neuropathic pain (2%) compared to the No group (16%), which was statistical significant (*p* = 0.039); whereas postoperative C2 hypoesthesia was significantly higher in Ex group (34%) compared to No group (6%) (*p* = 0.001).

There was one intraoperative CSF leak in the No group but none in the Ex group. There were no VA injuries recorded in either group. There was also no screw displacement in either group. The Fusion rate was very high (98%) in both group (*p* = 1.0). Detailed information about the outcomes is depicted in Table 3.

**Table 3:** Clinical characteristics and outcomes by surgical interventions

	Ex group	No group	*P*-value	Total
	No. Of Pts (%)	No. Of Pts (%)		No. Of Pts (%)
Surgery time in minutes, median [IQR]	214.0 [180.0, 277.0] **	174.0 [135.5, 241.0] **	0.001	199.0 [161.3, 250.0]
Bleeding in ml, median [IQR]	150.0 [100.0, 270.0]	200.0 [150.0, 300.0]	0.072	200.0 [100.0, 300.0]
Complications			0.184	
None	37 (74.0)	30 (58.8)		67 (66.3)
Minor	5 (10)	5 ( 9.8)		10 (10.0)
Major	8 (16.0)	16 (31.4)		24 (23.8)
Illiac bone harvesting			0.001	
Yes	6 (12.0) **	22 (43.1) **		28 (27.7)
No	44 (88.0) **	29 (56.9) **		73 (72.3)
Utilization of hard collar post-op (%)			< 0.001	
Yes	3 (6.0) **	19 (37.3) **		22 (21.8)
No	47 (94.0) **	32 (62.7) **		79 (78.2)
Mobilization (%)			0.049	
Day 1	43 (86) *	35 (72.9) *		78 (79.6)
Day 2	7 (14.0) *	6 (12.5) *		13 (13.3)
Day 3	0 (0.0) *	3 (6.2) *		3 (3.1)
No	0 (0.0) *	4 (8.3) *		4 (4.1)
Follow-up C2 neuropathy			0.039	
Yes	1 (2.0)	8 (15.7)		9 (8.9)
No	49 (98.0)	43 (84.3)		92 (91.1)
Follow-up C2 hypoesthesia			0.001	
Yes	17 (34.0) **	3 (5.9) **		20 (19.9)
No	33 (66.0) **	48 (94.1) **		81 (80.2)
Fusion			1.00	
Yes	49 (98.0)	50 (98.0)		99 (98.0)
No	1 (2.0)	1 (2.0)		2 (2.0)
Pre op ASIA A-E			0.65	
A	1 (2.0)	1 (2.0)		2 (2.0)
C	0 (0.0)	1 (2.0)		1 (1.0)
D	6 (12.0)	4 (7.8)		10 (9.9)
D/E	1 (2.0)	0 (0.0)		1 (1.0)
E	42 (84.0)	45 (88.2)		87 (86.1)
ASIA at discharge			0.35	
Improved	4 (8.0)	1 (2.0)		5 (5.0)
Unchanged	46 (92.0)	50 (98.0)		96 (95.0)
Post-op infection within 6 weeks (%)			0.5	
Yes	7 (14.0)	4 (7.8)		11 (10.9)
No	43 (86.0)	47 (92.2)		90 (89.1)
Re-op 3 months (%)			1.0	
Yes	4 (8.0)	3 (5.9)		7 (6.9)
No	46 (92.0)	48 (94.1)		94 (93.1)
Re-op 1 year (%)			0.37	
Yes	1 (2.0)	4 (7.8)		5 (5.0)
No	49 (98.0)	47 (92.2)		96 (95.0)
Follow-up cervial pain			0.31	
High	3 (6.0)	0 ( 0.0)		3 (3.0)
Low	8 (16.0)	8 (15.7)		16 (15.8)
Medium	3 (6.0)	5 (9.8)		8 (7.9)
None	36 (72.0)	38 (74.5)		74 (73.3)

^*^p < 0.05, chi-square test for heterogeneity, **p < 0.01, chi-square test for heterogeneity for categorical data and Mann Whitney U test for medians. Pt = patient

**Fig. 1:**
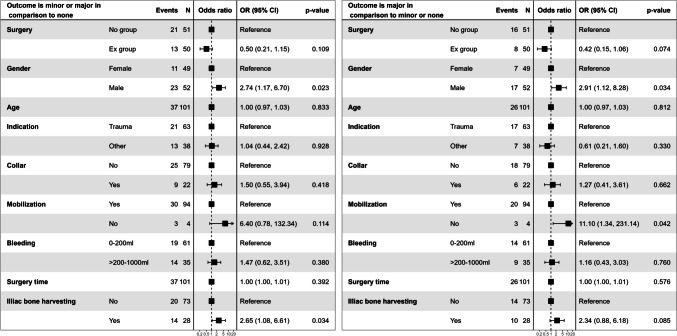
The forest plot with odds ratio of the development of minor and major complications versus major complications only in both the Ex and No groups

Male sex, delayed postoperative mobilization and bone harvesting from the iliac crest increased the OR for the development of minor and major complications in the postoperative period. However, only male sex and iliac bone harvesting were statistically significant. Further analysis demonstrated that delayed mobilization and male sex correlated significantly (*p* = 0.042 and *p *= 0.034 respectively) with higher OR of developing major postoperative complications. Iliac bone harvest also demonstrated higher OR but was not statistically significant. Detailed results are depicted in the forest-plot in Fig. 1.

## Discussion

Surgical treatment of pathologies in the cranio-cervical junction area can be technically challenging. It is mandatory that the choice of the surgical technique be considered on an individual basis and tailored to the specific requirements of the patient depending on the type of pathology, anatomy of C1 and C2 vertebrae, as well as the trajectory of screw placement, in order to avoid severe neuro-vascular complications. For example, types III and IV of C2 vertebrae, according to Tubbs classification, are not amenable to C2 pedicle instrumentation [33]. Utilization of image guidance can improve the precision of screw placement to reduce the risk for VA injury as well as support the choice of optimal screw trajectory to minimize misplacement and potential neurological injury [9, 21, 22]. Furthermore, it provides surgeons with intraoperative control of implant position. Hence, when executed correctly, C1/C2 fixation results in good clinical and radiological outcome [7, 15].

**Fig. 2:**
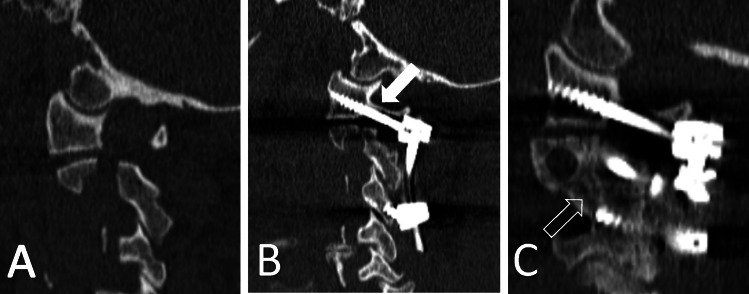
The example of a patient with C2 traumatic spondylolisthesis (**A**) operated with C1-C3 fixation with preservation of C2 nerve roots. Despite utilization of half-threaded screws, the space between C1 and C2 vertebrae was significantly constricted (white arrow), resulting in persistent C2 neuralgia (**B**). The follow-up images demonstrated fusion of C2 fracture (open arrow) but no visible fusion between C1 and C2 vertebrae (**C**)

The choice of C1/C2 instrumentation technique remains controversial. The Harms-Melcher technique has gained more popularity compared to the Goel-Laheri because it is technically easier and does not require sacrificing the C2 ganglion [11, 14]. It does, however, require manipulation of the C2 ganglion and proximal C2 nerve in order to gain access to the C1 lateral mass. Harms suggested the use of half-threaded screws for C1 instrumentation to avoid mechanical injury to the C2 nerve root. Two studies examined the outcome after C1/C2 instrumentation with preserved C2 nerve roots and concluded that it is a safe and efficient method for treatment pathologies in the cranio-cervical junction [26, 34]. Unfortunately, both studies overlooked the incidence of C2 postoperative neuralgia. Furthermore, in the majority of cases, a technique was utilized with screws being placed through the arch of the C1vertebrae, which is designed to avoid manipulation of the C2 nerve root and its plexus. Surgical manipulation of the C2 nerve root by itself can theoretically cause injury to the ganglion because it needs to be pulled down to expose the lateral mass. There is also a risk of excessive perioperative bleeding from the venous plexus surrounding the C2 nerve roots [7, 15, 26]. The bleeding can be quite severe, and more importantly obscures the exposure. Excessive use of bipolar cautery may cause additional thermal damage to the C2 ganglion. Furthermore, the mechanical manipulation of the C2 nerve root ganglion as it is stretched in a “bowstring” fashion under the inserted screw impinging on the ganglion may also be a potential mechanism of postoperative occipital neuralgia also referred as C2 neuropathy. Lu et al. investigated the size of the C1/C2 interspace and concluded that the C2 nerve-root ganglion occupies 76% of its entire height [24]. A correlation of the height between C1/C2 and development of C2 neuropathy was also demonstrated by Huang et al. [16]. This could explain the onset of postoperative neuralgia after instrumentation of the C1 vertebrae with preserved C2 nerve roots [4, 5, 12, 17, 29]. The example is illustrated in Fig. 2.

On the other hand, the scepticism towards the Goel-Laheri technique is based on concerns of the development of chronic postoperative C2 neuropathic pain due to nerve root transection. In a post-hoc analysis of prospective comparison study, Yeom et al. advocated against C2 nerve-root transection[35]. The authors described postoperative C2 neuralgia in 7 out of 24 patients (29%) after transection of the C2 nerve-root. However, in many cases the transaction was performed through the C2 ganglion using either bipolar with scissors or monopolar electrocautery. Furthermore, all C1 instrumentations in the preservation group were performed with placement of a C1 lateral mass screw directly through the arch of the C1 vertebrae. This technique is designed to avoid the dissection of C2 nerve-roots and the surrounding venous plexus. Hence, it can easily explain the fewer number of postoperative C2 neuralgia in 4 out of 41 patients (10%). In a recent prospective study by Singh et al., the authors compared two large cohorts, one with preserved C2 nerve-root and the other with transection of the C2 nerve-root. The surgical technique was not consistent because the C2 nerve root was incised sometimes proximally to the C2 ganglion but also through the C2 ganglion. Moreover, some cases planned for C2 nerve root preservation were converted to C2 nerve root incision due to the patient anatomy. Nevertheless, both groups presented with similar outcomes regarding C2 nerve root function. The authors concluded against scarifying C2 nerve roots because some of patients instrumented to the occiput developed a neuropathic ulcer in the region, which required surgical treatment with a vascular flap coverage or skin grafts [31]. This complication was previously described by other authors in approximately 5% of the patients operated with C2 nerve-root transection [30]. We postulate that this can be explained by postoperative hypoesthesia in the occipital region due to C2 nerve root transection in combination with postoperative use of hard collar and/or immobilization in bed. This could trigger skin irritation and abrasion due to constant pressure on the occipital region leading to deeper skin lesions. The literature review about the usage of hard collar supports our approach and demonstrates no statistically significant impact on the clinical results in patients treated with cervical fusion [19]. We did not encounter such healing problems in any of our patients, including the patients who developed postoperative infection and required surgical revision. This might also be explained by the fact that only a few patients in our study underwent instrumentation to the occiput and very few patients in Ex group required hard collar postoperatively.

Regarding the development of postoperative C2 neuralgia, our findings suggest differently. We encountered a eight times higher incidence of postoperative C2 neuropathy after preservation of the C2 nerve root compared to C2 ganglion resection and the difference was statistically significant. All the patients described this as very unpleasant and difficult to cope with, despite treatment with neuromodulation medications. The only case of postoperative C2 neuropathy in the Ex group, was an elderly patient with longstanding neck pain who sustained both axial and subaxial fractures that both required surgical treatment. The patient was unable to specify the character of the pain and, in general, did not fulfil the C2 neuropathy criteria but responded positively to neuromodulation pain management. Hence, we decided to treat it as C2 neuropathy. Our results corresponds to the findings published by Aryan et al., in their large retrospective case series study they encountered only one case (< 1%) of postoperative neuralgia after C2 nerve root trasection [2]. On the contrary, many more patients developed C2 neuralgia in the Yeom et al. and Singh et al. studies [31, 35].

We are convinced that the location of the incision of the C2 nerve root plays a crucial role, and that the injury to the C2 ganglion itself can be affiliated with development of occipital neuralgia. Hence, to mitigate the risk for C2 postoperative neuralgia, we have modified the Goel-Laheri technique, by conducting the incision both proximal and distal to the C2 ganglion, thus removing it completely. Furthermore, complete resection of the ganglion provides access not only to the C1/C2 joint but also to the remaining deep venous plexus, which can be coagulated or packed. This can explain why we encountered far less perioperative bleeding than described in the literature [2, 31, 32]. We reviewed the literature for comparison and to our knowledge, our is the only report of this technique since the common surgical practice is to divide the C2 nerve root proximally or directly through the ganglion [2, 5, 18, 30–32, 35]. Therefore, we strongly believe that our new surgical technique with complete resection of the C2 ganglia plays a role in our results. Moreover, it could explain the high frequency of postoperative hypoesthesia of the C2 dermatome, which was five times higher after the ganglion resection. However, none of the patients described it as bothersome or unpleasant. These findings correspond well with results presented by Dewan et al. where they investigated the impact of, respectively, C2 neuropathy and C2 anaesthesia on the quality of life. The authors concluded that postoperative anaesthesia in patients treated with C2 transection did not have any impact on the quality of life. On the contrary, the development of C2 neuropathy in patients with C2 nerve root preservation had an important negative impact on the quality of life [5].

Elliot et al. conducted a literature review to compare outcomes in patients with or without C2 nerve sacrifice during instrumented C1/C2 fusions [6]. However, the majority of the reviewed studies were small case series without a control group. The authors described that the incidence of neuropathic pain after planned C2 nerve root excision was negligible. On the contrary, instrumented C1/C2 fixations with preserved C2 nerve root, using the Harms-Melcher technique, resulted in nearly a 5% incidence of neuropathic pain of the C2 dermatome. Symptomatic numbness of the C2 dermatome was not clinically relevant but spontaneously reported in 12% of the patients. Moreover, several reports described open C2 nerve root transection as a valid option for treatment of intractable, medically resistant occipital neuralgia [1, 13, 23, 27].

Most studies describing the Goel-Laheri technique are small case series. To our knowledge, there are only four studies, two prospective and two retrospective, that use control groups in their analysis [5, 30, 31, 35]. Unfortunately in three of the studies, the cohorts are ill-matched with an asymmetrical number of patients in both groups. The strength of our study relies on its methodological comparison between two well-matched cohorts. Hence, based on our results, we perceive that our modified Goel-Laheri technique with excision of C2 ganglion has several important advantages. The first, and most obvious, is less perioperative bleeding than in the No group. Secondly, C2 ganglion excision diminished the requirement for iliac crest harvesting, postoperative hard collar, and significantly more rapid mobilization of patients. Not surprisingly, these factors contributed to significantly lower odds for development of postoperative minor and major complications in the Ex group.

**Fig. 3:**
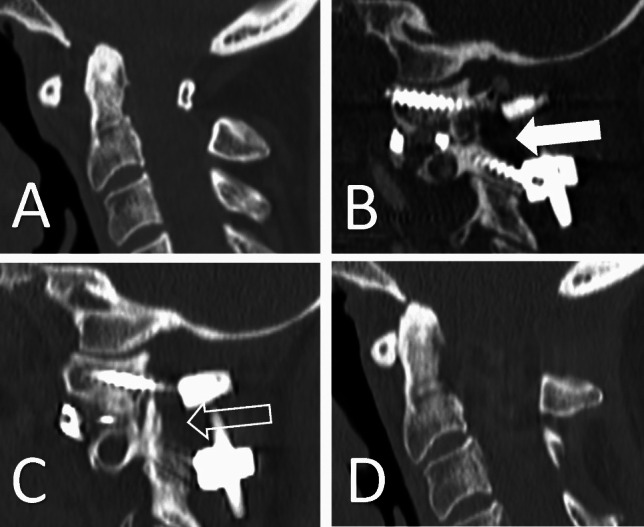
The example of a patient with rheumatoid arthritis with basilar impression and atlanto-axial instability (**A**). C1-C2 fixation with C2 nerve root resection was performed followed by reduction using intra-articular spacers filled with autologous bone (white arrow) (**B**). The follow-up images demonstrate solid fusion across C1/C2 joints (open arrow) (**C**) and successful reduction (**D**)

The fusion rates remained similar in both groups, albeit patients in the Ex group tended to develop fusion faster. This is because C2 ganglion resection enables full access to the C1/C2 joints, thus facilitating correction, instrumentation as well as favouring bone fusion with less amount of bone transplant. This is reflected by the significantly shorter radiological follow-up time. As mentioned previously, the majority of patients in the Ex group were operated on due to complex pathologies like cranio-cervical deformity or tumours. This is obviously selection bias related to the retrospective design of our study. However, this discrepancy between the Ex and No groups was advantageous toward the control group since almost half of the patients in the Ex group were operated on due to rheumatoid arthritis (RA) related complications. RA is a chronic inflammatory disorder that despite treatment can cause considerable damage to the upper cervical spine with atlanto-axial instability, development of pseudotumor and/or basilar impression. Thus, distorted anatomy with cranial settling caused by deformed C1 and C2 joints makes the access due to lateral masses technically challenging. Furthermore, osteoporosis, chronic use of cortisone and other immunomodulating agents make the RA patients extremely susceptible to postoperative complications like infections, non-union and hardware failure [3, 8]. The example of the basilar impression surgery from Ex group is illustrated in Fig. 3.

There was only one patient in the Ex group who required re-operation due to non-union and mechanical failure, whereas, there were four patients in the No group. Despite significant differences in diagnosis between the groups and the fact that the surgical time was significantly longer in Ex group the overall complication rate remained similar in both groups. Neurological outcome was also similar between the groups although more patients improved after surgery with C2 nerve root resection. More importantly, patients who underwent C2 nerve root resection demonstrated statistically significantly lower odds for development of postoperative minor and major complications due to lesser requirements for iliac-crest harvesting, usage of hard collar and faster postoperative mobilization.

## Conclusion

We conclude that excision of the C2 nerve root ganglion during instrumentation in the cranio-cervical region is a safe technique. It provides an additional exposure necessary for instrumentation with pedicle screws, but more importantly it minimizes bleeding from the venous plexus and allows the manipulation of the C1/C2 joints, which is often necessary for reduction of the fractures or deformities. Our results confirm that C2 neuropathic pain is more common after attempts to preserve the C2 nerve roots.

In our opinion, our modified Goel-Laheri technique with excision of C2 ganglion should be considered as an alternative technique in the treatment of complex pathologies in the cranio-cervical junction area, especially in cases of deformity and rheumatoid arthritis. This technique allows for early mobilization without hard collar and iliac crest harvesting, which seems to mitigate the risk of major postoperative complications. However, these results should be validated in a larger prospective randomized trial.

## Study limitations

This study has several limitations. The most important limitations are linked to the retrospective study design, which resulted in selection bias between the groups.

## Supplementary Information

Below is the link to the electronic supplementary material.

Supplementary file1 (DOCX 14 KB)

## Data Availability

No datasets were generated or analysed during the current study.
