# Supplementary material for: Safety and efficacy of the new modified technique for c2 nerve root resection in 3d fluoroscopy navigated instrumentation in the cranio-cervical junction
Source: Acta Neurochir (Wien). 2024 Sep 16;166(1):368. doi: 10.1007/s00701-024-06265-x (PMC11402833; doi:10.1007/s00701-024-06265-x)
Supplement: Supplementary file 1 — Supplementary file1 (DOCX 14 KB) [file 701_2024_6265_MOESM1_ESM.docx]

**Tables and figures legend:**

**Table 1.** Demographical characteristics by surgical interventions

**Table 2.** Type and extent of cervical fixations

**Table 3.** Clinical characteristics and outcomes by surgical interventions

**Figure 1.** The forest plot with odds ratio of the development of minor and major complications versus major complications only in both the Ex and No groups

**Figure 2.** The example of a patient with C2 traumatic spondylolisthesis (A) operated with C1-C3 fixation with preservation of C2 nerve roots. Despite utilization of half-threaded screws, the space between C1 and C2 vertebrae was significantly constricted (white arrow), resulting in persistent C2 neuralgia (B). The follow-up images demonstrated fusion of C2 fracture (open arrow) but no visible fusion between C1 and C2 vertebrae (C).

**Figure 3.** The example of a patient with rheumatoid arthritis with basilar impression and atlanto-axial instability (A). C1-C2 fixation with C2 nerve root resection was performed followed by reduction using intra-articular spacers filled with autologous bone (white arrow) (B). The follow-up images demonstrate solid fusion across C1/C2 joints (open arrow) (C) and successful reduction (D).
